# Wanted—An Isolation Hospital

**Published:** 1902-02

**Authors:** 


**A Monthly Review of Medicine and Surgery.**

*EDITOR:*

WILLIAM WARREN POTTER, M. D.

All communications, whether of a literary or business nature, books for review and exchanges should be addressed to the editor: 284 FRANKLIN STREET, BUFFALO, N. Y.

VOL. XLI.—LVII. FEBRUARY, 1902. NO. 7.

### Wanted—An Isolation Hospital.

AMONG the present needs of Buffalo that are urgent, in our view, quite the most important is an adequate isolation hospital with a detention annex. A city of the commercial importance of Buffalo,—the fifth port in the world and a great manufacturing center,—should not suffer for the want of any municipal action that would serve to advance its interests or protect its citizens. Especially should the inhabitants enjoy the best possible safeguards against epidemic catagious diseases.

It has been the boast of this city that its death-rate was the lowest in the world according to its population, a record that is due to the tireless energy of the former health commissioner, Dr. Ernest Wende, more than to any other one cause. But it will not answer for a great city to remain inactive, counting upon its record and resting upon its laurels. It must be progressive, active, alert, and indeed aggressive if need be in order to maintain its standing and rank in this restive world and impatient age. There is nothing so good that it ought not to be made better, nothing so perfect that it may not be improved.

It is with great satisfaction that we contemplate the efficiency of our health department, but every intelligent person knows that it must be supported by an enlightened public sentiment, as well as by ample pecuniary aid, in order to make its influence and scientific work available for the best results in the economics of public sanitation. No matter how energetic the officials of the department may be, the city must provide the necessary funds and equipment, to enable them to prosecute the work, else there will be shortcomings if not failures in results.

That a new modernly appointed isolation hospital is sorely needed in Buffalo, is known to most people here who are at all observant of the public needs. If any such remain to be convinced let them read what the present commissioner of health, Dr. Walter D. Greene, says in regard to the so-called quarantine station now in use in his paper on The Present Outbreak of Smallpox in Buffalo, printed elsewhere in this edition. The former health commissioner presented the subject over and again to the council, but no heed was paid to his appeals. Now Dr. Greene presents the matter in such a light, and his pen picture is so graphic, that to delay longer the construction of an adequate building will create a public scandal.

Let the council move at once and authorise the erection of a hospital under the direction of the health commissioner which shall combine an isolation hospital and a detention pavilion under one management. In this large port a suitable detention hospital should be provided where contagious suspects could be sent without detaining vessels under quarantine, pending the development of such cases.

